# The value of clinical-ultrasonographic feature model to predict the severity of secondary hyperparathyroidism

**DOI:** 10.1080/0886022X.2022.2027784

**Published:** 2022-02-15

**Authors:** Xiaoer Zhang, Wenxin Xu, Tongyi Huang, Jingzhi Huang, Chunyang Zhang, Yutong Zhang, Xiaoyan Xie, Ming Xu

**Affiliations:** Department of Medical Ultrasonics, The First Affiliated Hospital, Institute of Diagnostic and Interventional Ultrasound, Sun Yat-Sen University, Guangzhou, China

**Keywords:** Secondary hyperparathyroidism, nomogram, clinical-ultrasonographic feature, intact parathyroid hormone, contrast-enhanced ultrasonography

## Abstract

**Objectives:**

To analyze conventional ultrasound (CUS) and contrast-enhanced ultrasound (CEUS) features in patients with secondary hyperparathyroidism (SHPT) and to evaluate the clinical-ultrasonographic feature based model for predicting the severity of SHPT.

**Methods:**

From February 2016 to March 2021, a total of 59 patients (age 51.3 ± 11.7 years, seCr 797.8 ± 431.7 μmol/L, iPTH 1535.1 ± 1063.9 ng/L) with SHPT (including 181 parathyroid glands (PTGs)) without the history of intact parathyroid hormone (iPTH)-reducing drugs using were enrolled. The patients were divided into the mild SHPT group (mSHPT, iPTH <800 ng/L) and the severe SHPT group (sSHPT, iPTH ≥ 800 ng/L) according to the serum iPTH level. The clinical test data of patients were collected and CUS and CEUS examinations were performed for every patient. Multivariable logistic regression model according to clinical-ultrasonographic features was adopted to establish a nomogram. We performed K-fold cross-validation on this nomogram model and nomogram performance was determined by its discrimination, calibration, and clinical usefulness.

**Results:**

There were 19 patients in the mSHPT group and 40 patients in the sSHPT group. Multivariable logistic regression indicated serum calcium, serum phosphorus and total volume of PTGs were independent predictors related with serum iPTH level. Even though CEUS score of wash-in and wash-out were showed related to severity of SHPT in univariate logistic regression analysis, they were not predictors of SHPT severity (*p* = 0.539, 0.474 respectively). The nomogram developed by clinical and ultrasonographic features showed good calibration and discrimination. The accuracy and the area under the curve (AUC), positive predictive value (PPV), negative predictive value (NPV) and accuracy of this model were 0.888, 92.5%, 63.2% and 83.1%, respectively. When applied to internal validation, the score revealed good discrimination with stratified fivefold cross-validation in the cohort (mean AUC = 0.833).

**Conclusions:**

The clinical-ultrasonographic features model has good performance for predicting the severity of SHPT.

## Introduction

Secondary hyperparathyroidism (SHPT), characterized by overproduction of parathyroid hormone, is a serious complication of patients on maintenance hemodialysis for chronic kidney disease (CKD) [1]. Development and progression of SHPT may lead to various abnormalities, including increased bone and muscle pain, osteoporosis, pathological fracture, recurrent urinary stones, anemia, high blood pressure and neuromuscular disturbances. Furthermore, severe SHPT are associated with increased morbidity and mortality in CKD patients [2–4].

The diagnosis and the treatment decisions-making of SHPT are largely based on serum intact parathyroid hormone (iPTH) level [5]. Over secretion of iPTH has various adverse impact on skeletal and extra-skeletal tissue, may cause multiple symptoms and poor prognosis as mentioned before. Due to the potential affect in outcomes, iPTH level was recommended to be kept within a proper range [6]. For patients with CKD stage 5D and elevated iPTH level, calcitriol, or vitamin D analogs, or calcimimetics, or a combination of them are commonly used [7–9]. When severe SHPT develops and failed to respond to medical management, surgical parathyroidectomy (PTX) is generally considered [10,11]. Therefore, accurate evaluation is truly important in SHPT management.

Conventional ultrasound (CUS) and single photon emission computed tomography (SPECT) are commonly used in presurgical location of SHPT, and both of them show good accuracy in detecting parathyroid glands (PTGs) [12,13]. Nevertheless, considering the radioactivity of SPECT, CUS is the preferred inspection method for SHPT long-term follow-up due to its non-invasiveness, good reproducibility, real-time scanning and wide availability. Based on ultrasound contrast agent and harmonic imaging, contrast-enhanced ultrasound (CEUS) can reflect focal microcirculation dynamically and is gradually applied in SHPT examination. Besides, several studies have revealed that some ultrasound features of PTGs, both in morphology and vascularity, were correlated with serum iPTH level [14–17], that means ultrasound imaging may be a useful and relatively stable tool to evaluate severity of SHPT. However, the related research had no unified standard in grouping and grading, which attributed to some controversy. Besides, no final conclusion has yet been reached on which characteristics are independent factors for non-suppressible iPTH level, and no predictive model including both clinical and ultrasound parameters for evaluating severity of SHPT was established.

Therefore, the aim of this study was to analyze the CUS and CEUS features in patients with SHPT, construct a predictive model deriving form clinical-ultrasonographic features and investigate value of this model in evaluating severity of SHPT.

## Methods and materials

### Patients

This retrospective observational study was performed at The First Affiliated Hospital of Sun Yat-Sen University, approved by our institutional Ethics Committee. Ethnic approval code was [2019]219. Informed consent of every patient was not required. From February 2016 to March 2021, a total of 59 consecutive SHPT patients (age 51.3 ± 11.7 years, seCr 797.8 ± 431.7 μmol/L, iPTH 1535.1 ± 1063.9 ng/L, including 181 PTGs) were enrolled. CUS and CEUS were performed for every participant. The inclusion criteria were as follows: (1) patients with chronic kidney disease; (2) serum iPTH level above the upper normal limit on two or more occasions; (3) patients’ conditions remained stable during previous one month; (4) calcitriol, calcimimetic agent and vitamin D analogs were not used. Exclusion criteria included: (1) contrast agent allergy; (2) no parathyroid hyperplasia was identified in ultrasound imaging; (3) history of ablation or parathyroidectomy.

### CUS and CEUS examination

CUS and CEUS were carried out by an experienced sonologist with more than 5 years of experience in PTGs examination and CEUS preformation with one device. Toshiba Aplio 500 ultrasound scanner with a 5–14 MHz multifrequency linear probe (Canon, Tokyo, Japan) and Toshiba Aplio 900 ultrasound scanner with a 5–18 MHz multifrequency linear probe (Canon, Tokyo, Japan) were used. Patients were requested to keep supine position and extend neck as full as possible during scanning process. Basic B-scan and color Doppler were first used to identify and evaluate each suspected parathyroid gland. The basic B-scan was carried out from the left to right and corner of the jaw to the superior mediastinum of the anterior cervical with transvers, longitudinal and oblique scan to detect the parathyroid gland. Hypoechoic nodules behind thyroid gland in the anatomic location of parathyroid gland were considered as parathyroid gland recording their number, size, location, shape, and echo, and color Doppler would be performed to observe the blood supply of nodules. After CUS scanning, CEUS was subsequently implemented with a 2.4 mL suspension of six sulfur hexafluoride microbubbles (SonoVue, Bracco, Italy). Before bolus injection, the Sonovue powder was mixed with 5 mL saline solution and oscillated adequately. All injections were performed by the same assistant in all cases. Before CEUS, the target would be clearly showed on B-mode US and the probe was held steadily. Then sonologist got into CEUS mode with dual display picture under mechanical index (MI) 0.08–0.10. During the process of observation, which lasted for at least 3 min, patients were asked to breath quietly and slowly, avoid swallowing and speaking. The system would record the time since contrast-agent injection and sonologist would observe and record the enhanced pattern of nodules including the level of enhancement in early and late phase. CEUS was made for each suspected hyperplastic PTG assessed by CUS, and a new CEUS examination started only when previously injected contrast agent washed out completely.

**Figure 1.**
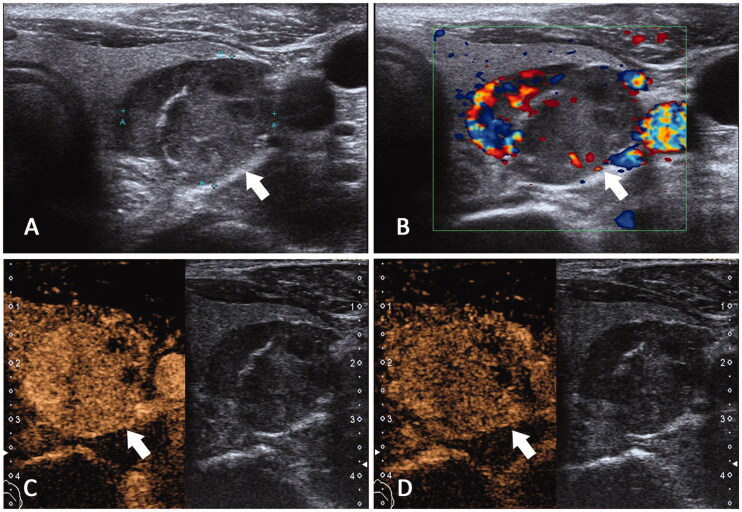
Ultrasonography feature of SHPT patient with high iPHT. A 55-year-old male diagnosed as SHPT for 8 years with high iPTH (3305pg/ml). (A) CUS detects a hypoecho lesion about 3.3 cm with calcification(white arrow). (B) Color Doppler showed the lesion has grade III blood supply. (C, D) In CEUS this lesion appeared to be hyper-enhanced in early phase and iso-enhanced in late phase.

**Figure 2.**
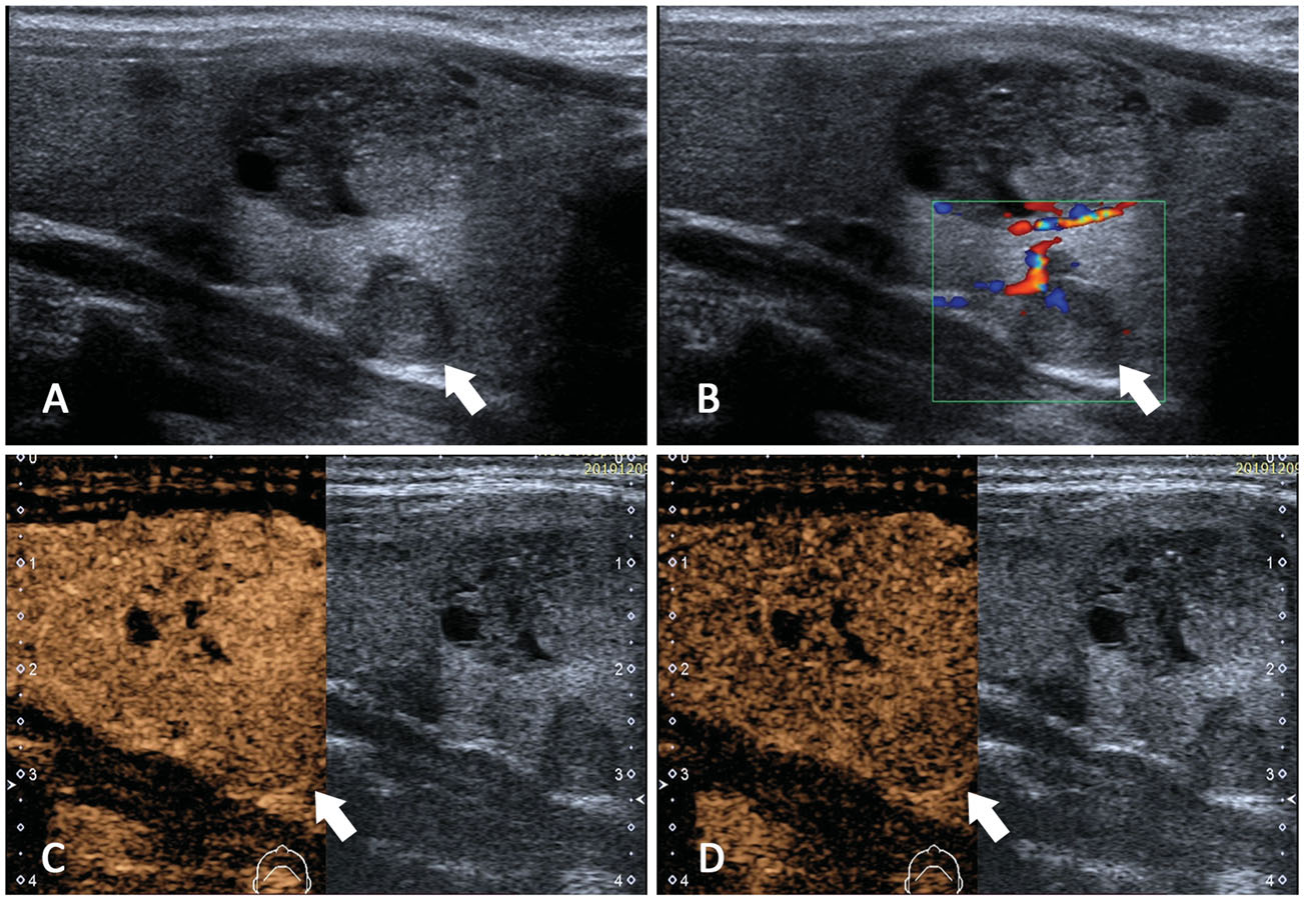
Ultrasonography feature of SHPT patient with low iPHT. A 52-year-old male diagnosed as SHPT for 4 years with low iPTH(443.5pg/ml). (A) CUS detects a hypoecho lesion about 1.0 cm (white arrow). (B) Color Doppler showed the lesion has grade I blood supply. (C,D) In CEUS, this lesion appeared to be iso-enhanced in both early and late phase.

All CUS and CEUS digital images were restored in picture or video format, and then analyzed by another two qualified sonologists, who were blinded to patients’ clinical information and laboratory findings. Blood supply was evaluated according to the Alder grading system of blood flow: grade 0 (absent): no blood flow was visualized; grade I (minimal): one- or two-pixels containing flow; grade II (moderate): a main vessel and/or several small vessels; grade III (marked): four or more vessels were observed [18]. The CEUS evaluation was composed of two phases: wash-in phase for 0–30s after the administration of contrast agent and wash-out phase for 30–120 s after contrast agent injection. Enhancement degree in wash-in and wash-out phase was evaluated as hypo-, iso- and hyper-enhanced (Figures 1 and 2). After imaging analysis, the ultrasound features including number of PTGs, localization, size, echo intensity, calcification, cysts, blood supply, vascular circle and enhancing pattern, were obtained and recorded.

### Study design

Because most patients had more than one hyperplastic PTG, and the number of PTGs was found to be correlated with the iPTH level [19], we put forward a scoring rule to evaluate the characteristics more roundly for a patient. Based on Alder grading system of blood flow, scores of different grades were set out below: 0, 1, 2 and 3 points for grades 0, I, II and III, respectively. By analogy, scores of different enhanced degrees in wash-in and wash-out phase were as follows: 1, 2 and 3 points for hypo-, iso- and hyper-enhanced, separately. Scores of all PTGs in one patient were accumulated to obtained the final blood supply score and enhancing score. As for calcification, cyst and vascular circle, similarly, we gave 0 point for inexistence and 1 point for existence, and accumulation of points of all lesions drawn total points for this patient. The volume of PTG was calculated in accordance with the formula *V* = (*a* × *b* × *c*) ×π/6 respectively, and then added up.

Except ultrasound examination, laboratory tests were performed once every 3 month for all patients and intervals between them were not exceeding one week. Serum calcium, phosphorus, potassium, creatinine and iPTH level were then obtained. Because therapeutic strategy for patients with SHPT is largely determined by the serum iPTH level and iPTH ≥ 800 ng/L is considered as an indication of PTX [20], we divided 59 patients with 181 lesions into two groups according to iPTH level: mild SHPT (mSHPT) group with iPTH < 800 ng/L and severe SHPT (sSHPT) group with iPTH ≥ 800 ng/L.

### Statistical analysis

All statistical analyses were performed using SPSS version 25.0 software (IBM Corp. Released 2017. IBM SPSS Statistics for Windows, Version 25.0. Armonk, NY: IBM Corp.) and R version 4.0.4 (https://www.r-project.org/). Distribution normality was assessed by the Shapiro–Wilk test. Continuous variables were presented as mean ± standard deviation (SD) or median (25th–75th percentiles) as appropriate. Differences between groups were compared with *t* test or Kruskal–Wallis rank sum test. Categorical variables were expressed as frequencies (percentages) and differences between groups were compared with the Chi-square test. The univariate and the multivariate logistic regression were performed to construct clinical-ultrasonographic feature derived model, based on which, a nomogram was established. Goodness of fit of this nomogram was tested by the Hosmer–Lemeshow test. Clinical utility of this nomogram was assessed by calibration curve and decision curve analysis (DCA). To evaluate the predictive value of this model, accuracy and the area under the curve (AUC) from a receiver operating characteristic (ROC) curve analysis was calculated. The AUC values of different models were compared by DeLong's test. All statistical tests were two-tailed and *p* < 0.05 was considered statistically significant. We also assessed generalizability using the mean AUC derived from stratified fivefold cross-validation.

## Result

### Clinical and ultrasonic characteristics

A total of 59 consecutive SHPT patients with 181 PTGs were enrolled finally, consisting of 32 men and 27 women. The average age and SD was 51.3 ± 11.7 years and average and SD of iPTH level was 1535.1 ± 1063.9 ng/L. The number of hyperplastic PTGs in each patient ranged from 1 to 6. But patients with 5 or 6 PTGs were relatively rare, only 3 (5.1%) and 1 (1.7%) respectively. About half of the PTGs had prominent vascularity, while the majority of PTGs had no calcification, cystic change or vascular circle.

There were 19 patients in the mSHPT group and 40 patients in the sSHPT group, separately. Serum phosphorus, creatinine, number of PTGs, total volume of PTGs, blood supply score, calcification and cyst score, CEUS score both in the wash-in and the wash-out phase in the sSHPT group were significantly higher than in the mSHPT group, while serum calcium was significantly lower (all *p* < 0.05). In terms of age, sex, serum potassium and vascular circle score, there were no significant difference between groups (all *p* > 0.05), as summarized in Table 1.

**Table 1.** Comparison of clinical and ultrasound features.

Variables	mSHPT group	sSHPT group	*p* Value
Clinical parameters			
Patients (*n*)	19	40	<0.001
Age (years)	52.2 ± 13.2	51.0 ± 10.7	0.714
Sex (male/female)	10 (52.6%)/9 (47.4%)	22 (55.0%)/18 (45.0%)	0.865
Calcium (mmol/L) (2.1–2.6 mmol/L)	2.6 (2.3–2.8)	2.4 (2.2–2.6)	0.037
Phosphorus (mmol/L) (0.97–1.62 mmol/L)	1.3 ± 0.6	2.0 ± 0.5	<0.001
Creatinine (μmol/L) (53–115 μmol/L )	118.0 (68.0–995.0)	954.0 (677.8–1158.0)	0.009
Intact parathyroid hormone (ng/L) (12.0–88.0 ng/L)	330.0 ± 241.6	2107.6 ± 783.0	<0.001
Potassium (mmol/L) (3.5–5.3 mmol/L)	4.2 ± 0.5	4.3 ± 0.8	0.531
Ultrasonic parameters			
Number of PTGs (n)	2 (1–3)	4 (2–5)	0.005
Total volume of PTGs (cm^3^)	0.8 (0.4–2.4)	3.3 (2.0–5.1)	<0.001
Blood supply score (point)	5 (2–9)	9 (5–12)	0.005
Calcification and cyst score (point)	0 (0–1)	1 (0–2)	0.038
Vascular circle score (point)	1 (0–2)	0 (0–1)	0.344
CEUS score (point)			
Washing-in phase	5 (3–8)	8 (6–11)	0.008
Washing-out phase	4 (3–6)	8 (5–10)	0.013

Continuous variables are expressed in mean ± standard deviation (SD) or median (25th–75th percentiles). Categorical variables were expressed as frequencies (percentages). mSHPT: mild secondary hyperparathyroidism; sSHPT: severe secondary hyperparathyroidism; PTG: parathyroid gland; CEUS: contrast-enhanced ultrasound.

### Univariate and multivariate binary logistic regression analyses

Clinical and ultrasound features with *p* < 0.05 in univariate logistic regression analysis were selected into multivariate logistic regression analyses. Results (Table 2) showed that serum calcium (*p* = 0.032, OR = 0.004), phosphorus (*p* = 0.029, OR = 22.542) and total volume of PTGs (*p* = 0.022, OR = 2.853) were independent factors for predicting severity of SHPT.

**Table 2.** Univariate and multivariate binary logistic regression analyses of predictors for severity of SHPT.

	Univariate analysis		Multivariate analysis	
Variables	*p* Value	OR (95%CI)	*p* Value	OR (95%CI)
Clinical parameters				
Age (years)	0.708	0.991 (0.946–1.039)		
Sex (male/female)	0.865	0.909 (0.304–2.717)		
Calcium (mmol/L)	0.023	0.079 (0.009–0.706)	0.032	0.004 (0.000–0.620)
Phosphorus (mmol/L)	<0.001	9.835 (2.932–32.987)	0.029	22.542 (1.382–367.630)
Creatinine (μmol/L)	0.002	1.002 (1.001–1.004)	0.144	0.997 (0.993–1.001)
Potassium (mmol/L)	0.525	1.286 (0.592–2.791)	–	–
Ultrasonic parameters				
Number of PTGs	0.013	1.673 (1.071–2.616)	0.873	1.170 (0. 171–7.990)
Total volume of PTGs	0.006	1.075 (1.021–1.131)	0.022	2.853 (1.164–6.993)
Blood supply score	0.008	1.261 (1.060–1.501)	0.717	1.090 (0. 685–1.734)
Calcification and cyst score	0.053	1.772 (0.992–2.987)	0.428	0.574 (0.146–2.262)
Vascular circle score	0.463	0.809 (0.460–1.423)		
CEUS score				
Washing-in phase	0.010	1.294 (1.064–1.575)	0.539	1.550 (0. 383–6.270)
Washing-out phase	0.017	1.276 (1.045–1.558)	0.474	0.608 (0.156–2.372)

SHPT: secondary hyperparathyroidism; PTG: parathyroid gland; CEUS: contrast-enhanced ultrasound.

### Development of predictive nomogram model for severity of SHPT

**Figure 3.**
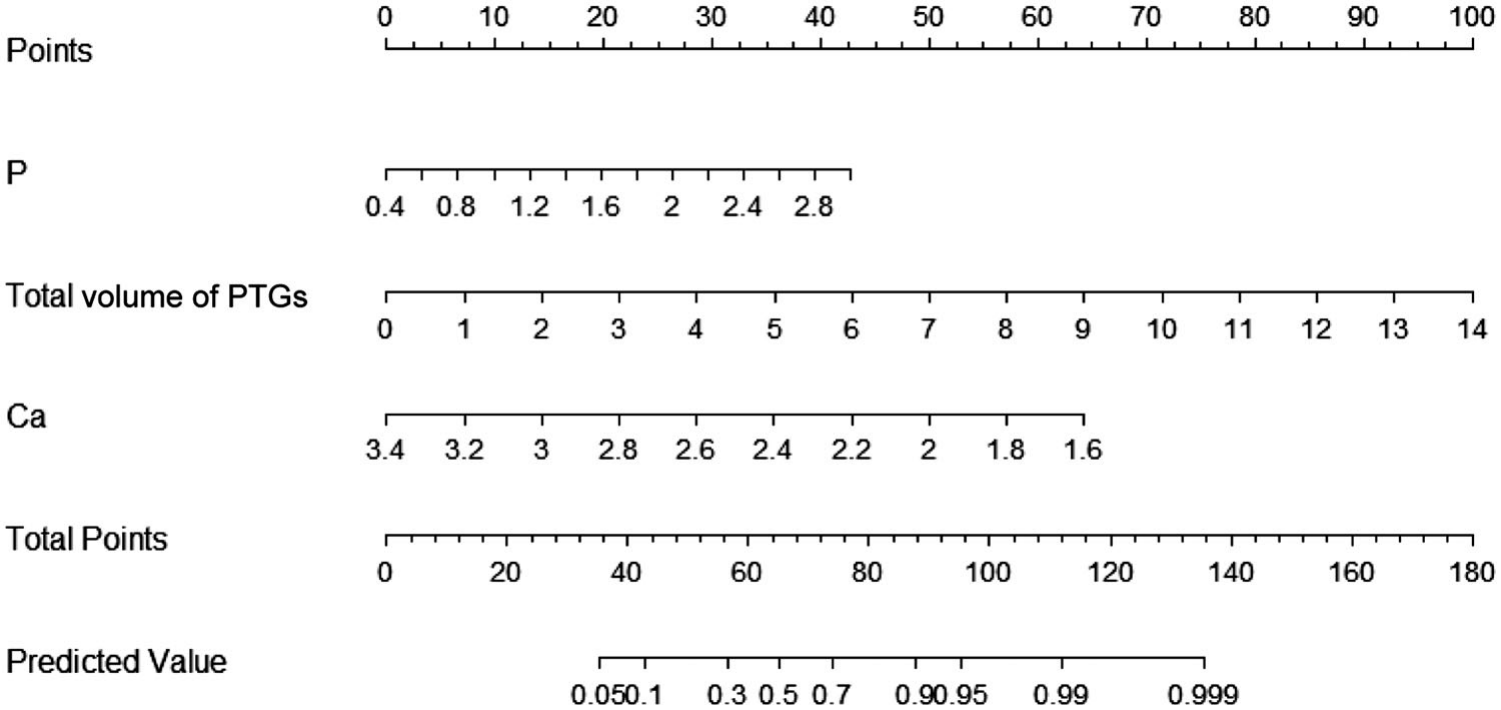
Clinical-ultrasonographic nomogram to predict severity of SHPT.

To display this predictive model more institutively, a nomogram with three factors as previously mentioned was constructed (Figure 3). Matching points of every parameter in the nomogram would be added up to obtain a total score, based on which the severity of SHPT was evaluated. For example, a patient with 2.4 mmol/L (35 points) serum calcium, 1.6 mmol/L (20 points) serum phosphorus and 5.0 cm^3^ (35 points) total volume of PTGs would score a total of 90 points, which corresponding to >90% predictive risk and meant strong intendency for severe SHPT.

**Figure 4.**
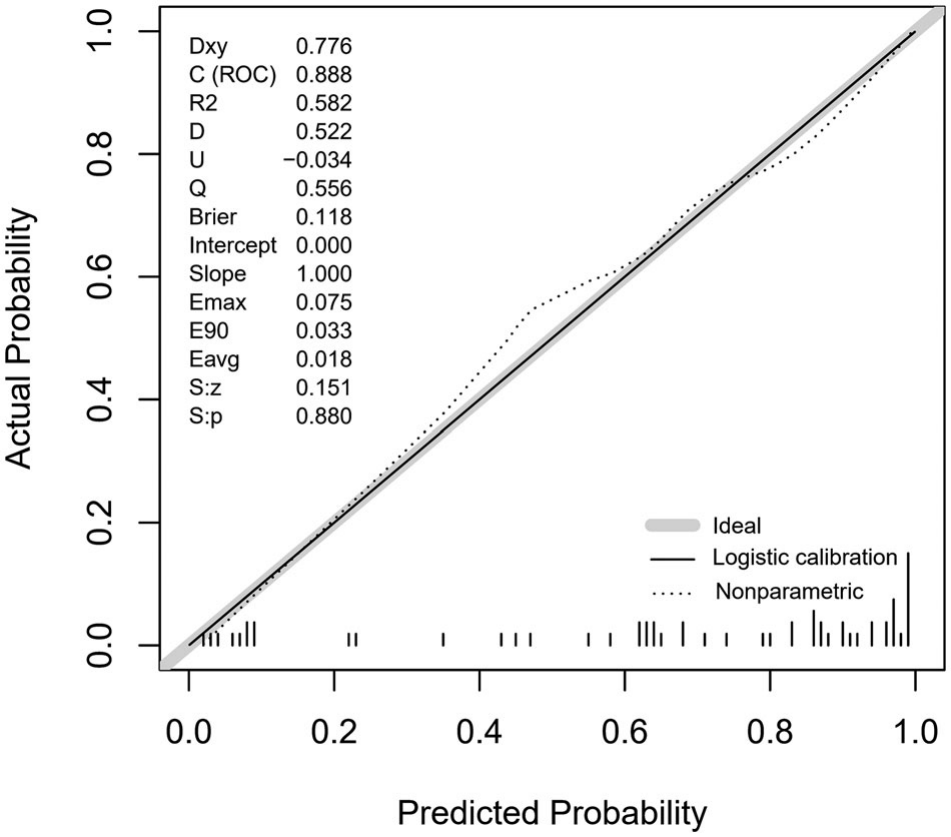
Calibration curves of the clinical-ultrasonographic nomogram.

Hosmer–Lemeshow test’s *p*-value was 0.949, indicating a satisfactory goodness-of-fit. The calibration curve is demonstrated in Figure 4, which implied that the predicted severity of SHPT came from nomogram was basically consistent with reality.

**Figure 5.**
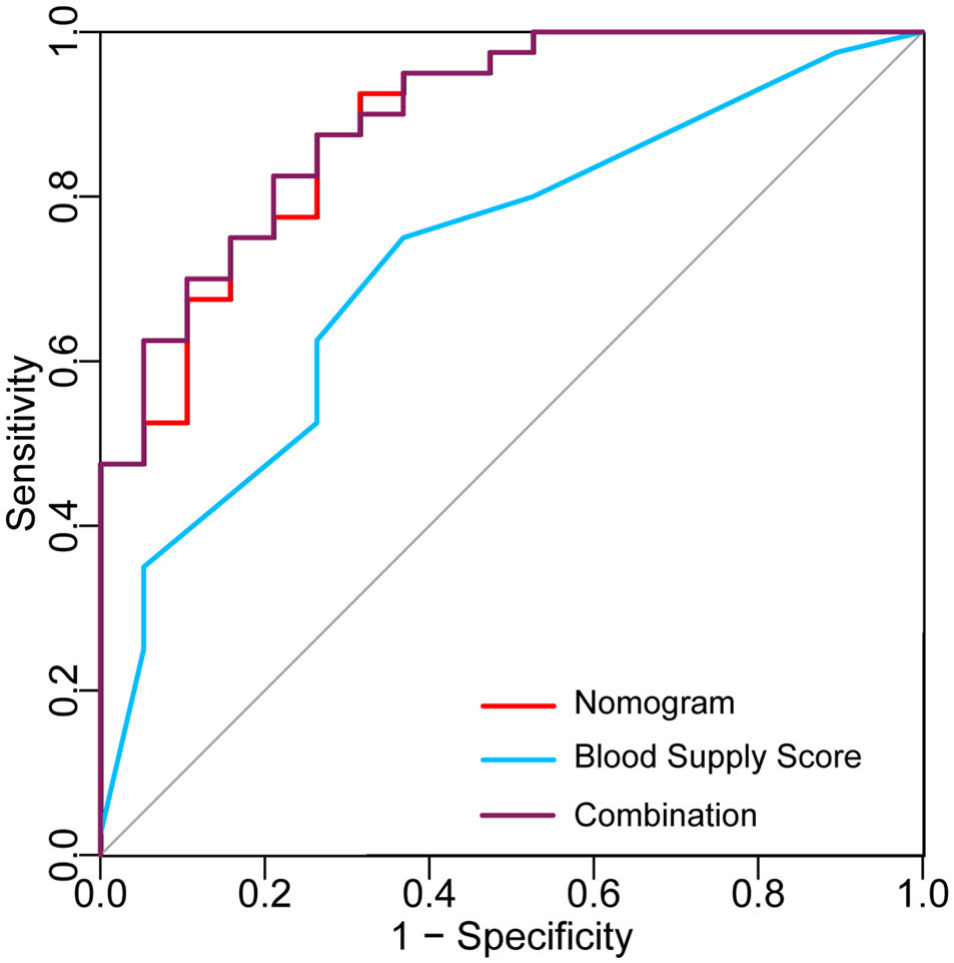
ROC curve analyses of the clinical-ultrasonographic nomogram and blood supply score combined with nomogram to compare the predictive performance.

Discriminatory capacity and generalizability were determined through stratified fivefold cross-validation in the cohort (AUC =0.943, 0.727, 0.781, 0.886, 0.821; mean AUC = 0.832). The ROC curves result showed that the AUC of nomogram was 0.888 (95% CI, 0.801–0.975) (Figure 5). When choosing the prediction of 0.627 as optimal cutoff value, corresponding sensitivity and specificity were 87.5% and 73.7%. The positive predictive value (PPV), negative predictive value (NPV) and accuracy of this model were 92.5%, 63.2% and 83.1%, respectively. AUC value of blood supply score was 0.725, significantly lower than that of nomogram (*p* = 0.035). While no significant difference was found when the AUC value of the combination of nomogram and blood supply score compared with the nomogram (0.896 versus 0.888, *p* > 0.05).

**Figure 6.**
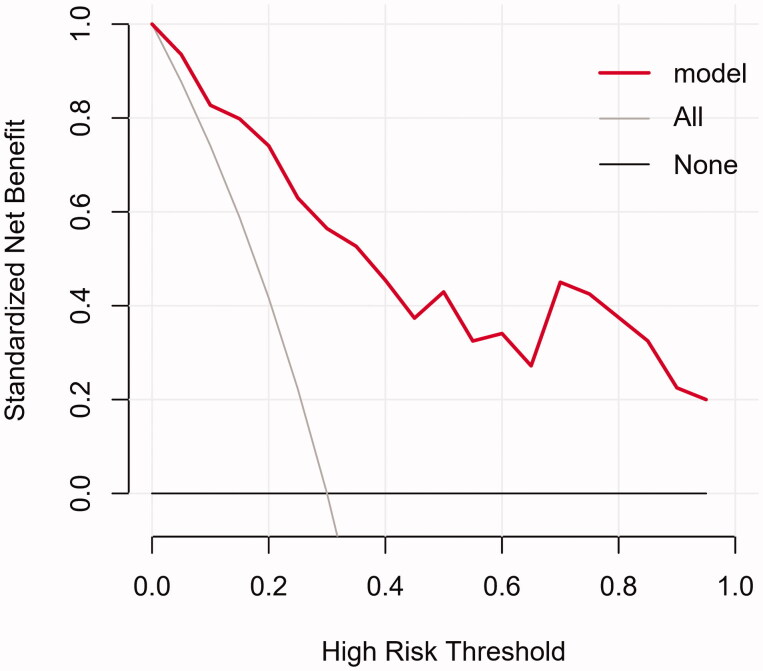
DCA for the clinical-ultrasonographic nomogram.

The DCA is shown in Figure 6, in which the nomogram performed well and was feasible to make beneficial clinical decisions.

## Discussion

In this study, we developed a clinical-ultrasonographic feature based model in helping to evaluate the severity of SHPT, including serum calcium, serum phosphorus and total volume of PTGs. The model showed good calibration and discrimination. And the AUC, PPV, NPV and accuracy of this model were 0.888, 92.5%, 63.2% and 83.1%, respectively.

In the present study, inter-relationship between iPTH level and other minerals’ concentration were explored. Serum calcium and phosphorus were found being independent indicators for evaluating severity of SHPT. The calcium-sensing receptors (CaSR) on the surface of chief cells in PTGs are sensitive to variation of serum calcium, that is how hypocalcemia leads to hypersecretion of iPTH [21,22]. Meanwhile, serum phosphorus attributing to decreased renal function triggers fibroblast growth factor 23 (FGF23) production, which inhibits 1 to 25-dihydrovitamin D3 (calcitriol) synthesis, and therefore causes iPTH overproduction and PTG cells proliferation [23–25]. Several studies indicated SHPT would be more severe when serum phosphorus level increased [14,26], which was consistent with our result. In our study, serum phosphorus in mSHPT group was significantly lower than that in sSHPT group (1.3 ± 0.6 mmol/L versus 2.0 ± 0.5 mmol/L, *p* < 0.001), and serum phosphorus was independently related with iPTH level. In terms of serum calcium, Indridason et al. [15] reported that iPTH level increased with the reduction of serum calcium concentration. But Wan et al. [27] reported inverse results. In their study, higher serum calcium level was observed in the group with higher iPTH level. While Vulpio et al. [19] found that serum calcium level was similar between the moderate and the severe SHPT group. In another study, serum calcium was found correlated negatively with iPTH level with statistical significance in simple regression but lost this correlation in multiple regression [14]. By this token, predictive value of serum calcium seemed to be disputable. In our study, serum calcium concentration in the mSHPT group was significantly higher than that in the sSHPT group (2.6 mmol/L versus 2.4 mmol/L, *p* = 0.037), and it was one of the independent predictors of iPTH level as well. As regard the discrepancy of serum calcium, we supposed that hypocalcemia is easier to be rectified than hyperphosphatemia may be one of the reasons. In consequence, when patients were grouped according to different criteria, difference of serum calcium level between groups may be not always as significant as phosphorus. To sum up, previous studies combined with ours indicated that serum calcium and phosphorus concentration should be both concerned, especially the latter. Patients with lower serum calcium and higher serum phosphorus were more likely to have severe SHPT.

Total volume of PTGs, connected directly with number and diameters of hyperplastic PTGs, was important indeed for predicting severity of SHPT. It is well known that over-proliferation of PTG cells leads to hyperplasia of PTGs, which is the histological foundation of SHPT. Greater total volume of PTGs therefore indicates the existence of more chief cells capable of producing and secreting iPTH. Detected PTGs number was reported to be significantly different between different SHPT degrees. Besides, maximum longitudinal diameter (MLD) and volume of the largest PTG was found to be related with SHPT severity [19,28–30]. Some studies also implied that total volume of PTGs showed close correlation with serum iPTH level [1,15]. Those results were basically unanimous with our study. Most of patients (93.2%) in present cohort had 1–4 PTGs, in accord with previous studies [16,31]. As expected, the total volume of PTGs in sSHPT group was higher than that in the mSHPT group with statistical significance (*p* < 0.001), and it was also found to be one of the independent indicators for predicting SHPT degree. Consequently, all diameters in three-dimensional of PTGs should be measured and every PTG should be taken into account.

There were some other different CUS and CEUS features between mSHPT and sSHPT groups, including vascular pattern, calcification and cyst and enhanced pattern. Some previous studies reported that correlation existed between vascularity and PTGs volume [32,33], but no evidence that suggests correlation between vascular pattern and SHPT degree had been found. Until Vulpio et al. [19] proposed a relatively complex grading standard to semi-quantify the vascular pattern of PTGs, vascularity of the largest PTG was discovered related with iPTH level. In our study, the Alder grading system, a semiquantitative method that often used in evaluation of breast tumors, was adopted to characterize the vascularity of PTGs. About half of PTGs (51.4%) were rich in blood supply, while mild and moderate blood flow signals were respectively observed in 41(22.6%) and 47(26.0%) PTGs. Logistic regression analysis revealed that blood supply score was a significant predictor of severity of SHPT, but not independent. As for calcification and cyst, and enhanced pattern of CEUS, both of them were significantly different between groups in accordance with limited previous studies [33,34]. Nevertheless, in our study, they were not independently related with iPTH level as well, like vascularity. Besides, Qin et al. [35] used CEUS to guide radiofrequency ablation therapy, which can assist the surgeon in making an accurate assessment both before and after the procedure.

The AUC of nomogram was 0.888, which is greater than 0.8, indicating satisfactory predictive performance of this model. In addition, to further determine the value of blood supply score, ROC analyses of this parameter and the nomogram combined with it were performed. AUC value of nomogram was significantly better than that of blood supply score, and similar to the combination of both of them, indicating that blood supply score was of less effectiveness in improving the efficiency of nomogram.

In present study, we chose iPTH level of 800 ng/L to be critical value, which met requirements of clinical management better. Furthermore, we firstly determined independent predictors of severity of SHPT and highlighted the clinical and ultrasound indicators that should be of greater concern, including serum calcium, serum phosphorus and total volume of PTGs. The corresponding nomogram was constructed and eligible in clinical practice, according to the satisfactory results of the Hosmer–Lemeshow test and DCA analysis.

There were some limitations of the present study. First, as a retrospective observational research, selection bias was inevitable. Second, our sample number was small and all enrolled patients came from a single center. Third, because of the limited sample number, further research needs to be done to verify this predictive model.

## Conclusion

Serum calcium, serum phosphorus and total volume of PTGs were found to be independent predictors of serum iPTH level in CKD patients without suppressing therapy, revealing that ultrasonography combined with laboratory parameters can help to evaluate the severity of SHPT more accurately. Besides, further large-scale researches are needed to validate present results.
